# Two classes of nucleic acid translocation motors: rotation and revolution without rotation

**DOI:** 10.1186/2045-3701-4-54

**Published:** 2014-09-16

**Authors:** Peixuan Guo, Ian Grainge, Zhengyi Zhao, Mario Vieweger

**Affiliations:** Nanobiotechnology Center, Markey Cancer Center, Lexington, KY USA; Department of Pharmaceutical Sciences, College of Pharmacy, University of Kentucky, Lexington, KY 40536 USA; Biological Sciences, School of Environmental and Life Sciences, University of Newcastle, Callaghan, 2308 Australia

**Keywords:** Bacteriophage phi29, Hexameric ATPase, Biomotor, Revolution, DNA translocase, DNA helicase, Viral assembly, DNA packaging

## Abstract

Biomotors are extensively involved in biological processes including cell mitosis, bacterial binary fission, DNA replication, DNA repair, homologous recombination, Holliday junction resolution, RNA transcription, and viral genome packaging. Traditionally, they were classified into two categories including linear and rotation motors. In 2013, a third class of motor by revolution mechanism without rotation was discovered. In this issue of “Structure and mechanisms of nanomotors in the cells”, four comprehensive reviews are published to address the latest advancements of the structure and motion mechanism of a variety of biomotors in archaea, animal viruses, bacteria, and bacteriophages.

## Main text

Nucleic acid translocation is ubiquitous in living systems. The motion required for these events is accomplished by biomotors hydrolyzing nucleotide (mainly ATP). Biomotors were once classified into two categories: linear and rotation motors. Accordingly, linear motors such as kinesin and myosin, seen in human and animal muscle coordination, move linearly, while rotation motors such as DNA helicases, F1/F0 ATPase, and bacterial flagella induce motion through a nut and bolt rotation mechanism. This concept of rotation for DNA translocation has been well-accepted in the field of biomotors due to the helical nature of the DNA with a 360° turn per pitch. The hypothesis that DNA packaging in dsDNA viruses was accomplished by such a five-fold/six-fold rotation motor survived for three decades. Numerous papers have been published in many high profile journals claiming the observation of five-fold viral motors with a rotation mechanism. However, attempts to evaluate this popular rotation mechanism has led to a wealth of contradictory experimental results. Gearing of the phi29 motor by a pRNA hexameric ring was revealed independently by both group of Peixuan Guo [1] and Dwight Anderson in 1998 [2], and highlighted in a minireview in *Cell* by the open-minded and visionary scientist Roger Hendrix [3], who even was the originator of the five-fold/six-fold rotation concept. In addition, biotechnological and single-molecule experiments revealed that neither the nut (motor channel/the viral connector) nor the bolt (dsDNA genome) rotate during packaging, thus, the rotation mechanism became an enigma.

**Figure 1:**
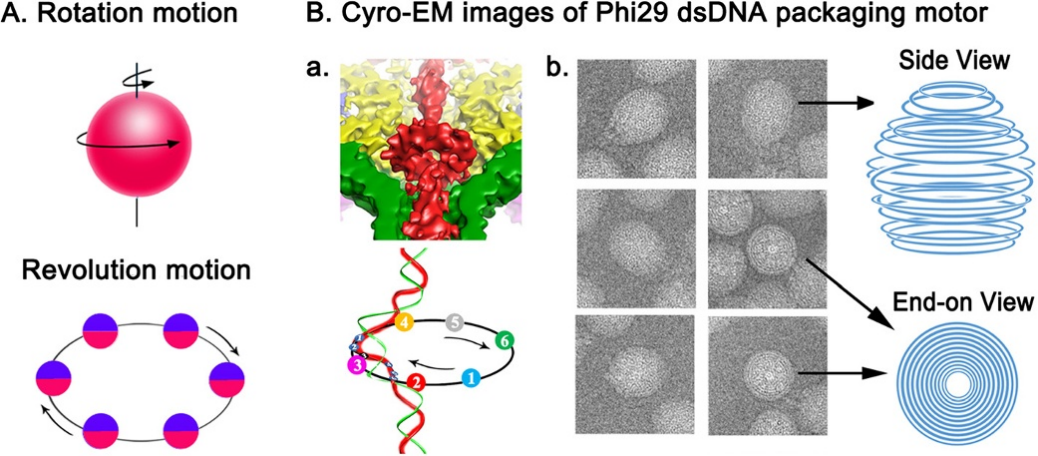
**Illustration of revolution versus rotation motion.** Spooling of the genome inside the procapsid supports the revolution mechanism. **A**. Rotation resembles the Earth’s motion about its axis once every 24 hours, while revolution resembles the Earth ‘circling’ around the Sun once every 365 days (see animations: http://nanobio.uky.edu/movie.html). **B**. **(a)** Cyro-EM images of phi29 particles in a cut-away surface showing the formation of a empty DNA toroidal ring. Due to the persistence length of dsDNA, which is around 150 bp, it is impossible for dsDNA to form a ring with a radius of only 0.9-2.9 nanometers without wrapping around protein. Since Cryo-EM picture is the average of many images, the toroid formation could be resulted from a collective image of many dsDNA revolving steps. **(b)** Cyro-EM images showing the spooling motion of phi29 genome during packaging as a result of the revolution motion. (Figure 1A: Adapted from [8] with permission of *BioMed Central*; Figure 1B: **a**. Adapted from [8] with permission of *BioMed Central* and from [9] with permission of *Elsevier*; **b**. Adapted from [10] with permission of *Elsevier*).

**Figure 2:**
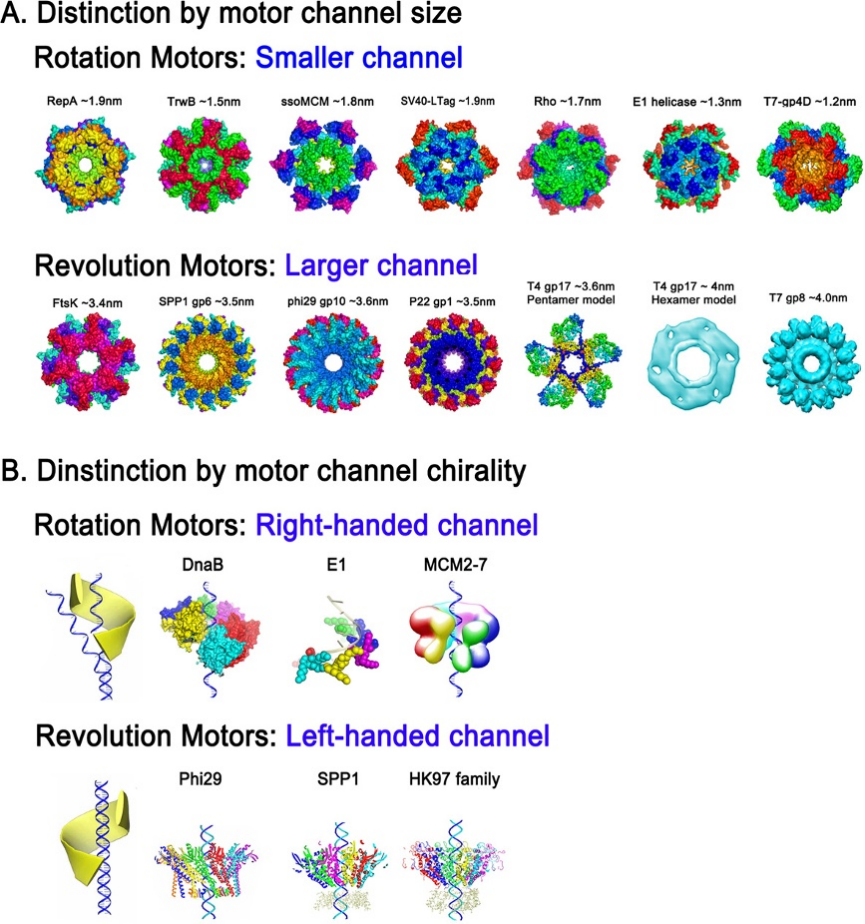
**Motor channel size and chirality as two distinguishing features of revolution and rotation motors. A**. Rotation motors have a channel size smaller than 2 nm to allow full contact between channel wall and the single-stranded nucleic acid inside the channel. Revolution motors have channel sizes larger than 3 nm to provide enough space for the revolution motion. **B**. Rotation motors use a right-handed channel to drive the right-handed DNA through parallel threads, while revolution motors use a left-handed channel in an anti-chiral arrangement with the right-handed dsDNA during translocation. (Adapted from [8] with permission of *BioMed Central, and adapted from*[11]*with permission from Elsevier*). (PDB IDs: RepA, 1G8Y; TrwB: 1E9R; ssoMCM, 2VL6; SV40-LTag, 1SVL; Rho, 3ICE; E1, 2GXA; T7-gp4D, 1E0J; FtsK, 2IUU; SPP1-gp6, 2JES; phi29-gp10, 1H5W; P22-gp1, 3LJ5; T4-gp17, 3EZK; DnaB, 4ESV; HK97 family-portal protein, 3KDR. EM IDs: T7-gp8, EMD-1231; MCM, EMD-1834).

In 2013, a third class of motor employing a revolution mechanism without rotation was reported (see animations: http://nanobio.uky.edu/movie.html) [4–6] (Figure 1). While rotation involves spinning of an object around its own axis, revolution is the circular movement of an object around a secondary center-object. By analogy, rotation resembles the Earth’s motion about its axis once every 24 hours, whereas revolution resembles the Earth ‘circling’ around the Sun once every 365 days (Figure 1). More recently, the revolving biomotor was found to be widespread among many biological systems, including dsDNA viruses, dsDNA bacteriophages, and bacteria [7, 8]. The rotation and revolution mechanisms can be distinguished by size and chirality of the motor channels [7, 8] (Figure 2). In most, if not all, rotation motors, only one strand of the DNA passes through the channel, however, both strands are translocated within the channel of revolution motors [4–8]. Rotation motors, such as helicases CMG, DnaB and E1, operate with a right-handed channel to drive the right-handed dsDNA. Parallel threads of channel and DNA allow sliding of the nut (channel) over the bolt (DNA) during the translocation process. Revolution motors, such as FtsK and the dsDNA packaging motors of phi29, P22, T4, HK97 and T3, use a left-handed channel to drive the right-handed DNA in an anti-chiral arrangement [7, 8]. Anti-parallel threads of channel and dsDNA facilitate step-wise advancement of the DNA by revolving during translocation through subsequent contacts between channel subunits and one strand of the dsDNA. The explicit difference between these packaging motors is further substantiated by the observation that rotation motors exhibit channel sizes equal to or smaller than 2 nm in diameter compared to channels larger than 3 nm in revolution motors (Figure 2) [7, 8]. Considering the diameter of the dsDNA helix of 2 nm, this structural feature agrees well with the distinction of the two mechanisms. In revolution motors, both strands of DNA pass through the channel, hence the diameter needs to be larger than 2 nm to ensure sufficient space for the dsDNA to revolve. In rotation motors, only one strand passes through the channel to ensure close contact between channel and ssDNA. Thus, the channel needs to be smaller than 2 nm, the diameter of the dsDNA helix.

**Figure 3:**
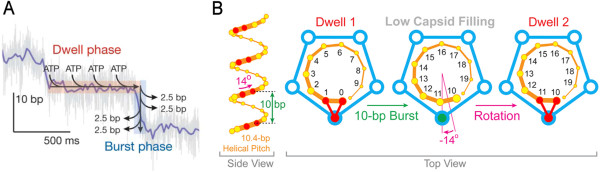
**Information showing the deeply rooted concept of the seemingly five-fold rotation in viral DNA packaging motors proposed several decades ago.** This concept is still found in recent publications proposing a five-fold rotational phi29 DNA packaging motor (both Figure 3A and B) [12–14]. The phi29 DNA packaging motor used in this figure is the same as constructed by Guo in 1986 [17, 18], who also discovered in 2013 the revolution mechanism of the phi29 motor [4–8] (see Figure 1, which is completely different from the five-fold rotational model). In Figure 3A, it was reported that the motor rotates in four steps [18, 19], contradictory to the traditional five-fold concept and thus requiring the supposition that one of the five subunits is inactive, resulting in a motor with only four steps of rotation [13, 14]. Interestingly, to maintain the rotation motor concept, the authors reported 1.5° rotation per bp, which corresponds to just 15.75° per complete helical turn of 10.5 bp translocated (Figure 3B) [12], while incompatible to the reality that 10.5-bp of DNA is a 360° helical pitch. (**A**: Adapted from [13] in *Nature* with permission of *Nature Publishing Group*; **B**: Adapted from [12] in *Cell* with permission of Elsevier).

Although use of the revolution mechanism without rotation in the bacteriophage phi29 DNA packaging motor has been clearly documented by several recent publications [4–8], the deeply rooted rotation concept has led to continued publications on the five-fold rotation mechanism [12–14] (Figure 3). A very recent paper claimed that the phi29 DNA packaging motor is a rotation motor with the observation of a 1.5° rotation per bp (that is 15.75° per helical turn of 10.5 bp) during DNA packaging [12] (Figure 3). This report is contradictory to the fact that packaging of one complete turn of dsDNA with a 10.5 bp pitch by a rotatory motor would require a rotation of 34° per bp instead of the reported 1.5°. Hence the mechanism is clearly not that of a classical rotary motor that follows the helical backbone, since packaging of a 360° helical turn could not have induced only a 15.75° twist. How nature resolves such a huge mismatch between 15.75° and 360° in a spatial configuration raises another big puzzle to the scientific community. This report further stands in contrast to a recent study examining the energy requirement of untwisting and twisting during motor rotation, which concludes that larger than four times the energy available from one ATP hydrolysis would be required [6, 15]. Actually, the small angular twist per nucleotide can be associated with channel conformational changes based on experimental data from cryo-EM [9]. At the early stages of packaging, the channel exhibits a left-handed conformation allowing the DNA genome to enter the viral procapsid. This left-handed conformation may need to be converted to right-handed at the completion of packaging to prepare for dsDNA ejection into the host cell [8]. As the dsDNA is aligned with the wall of the connector, it exhibits a clockwise twist associated with the channel conformational rearrangements [8]. It has also been proposed that in order to keep the dsDNA substrate in register with the motor during revolution, a small amount of twist of protein or DNA may be necessary [16, 17]. This might account for the 1.5° twist reported by Liu et al. [12].

As mentioned above, biomotors are ubiquitous in living systems and share many commonalities. In this special issue of Cell & Bioscience, the molecular mechanisms as well as biochemical and structural properties of a wide range of biomotors involved in archaeal DNA repair, dsDNA virus replication, viral and cellular SOS response, and double Holliday junction dissolution are comprehensively reviewed [20–23]. She et al. illustrate the structural analyses of archaeal nucleic acid biomotors in DNA damage repair and the molecular mechanisms of ATP hydrolysis promoting conformational change as the driving force of mechanical motion. Butcher et al. discuss a wide range of ATPases in the lifecycle of thermophilic archaeal dsDNA viruses from a bioinformatical, biochemical and structural point of view. This review in particular illustrates, how Sf2 helicases translocate along DNA in the classic fashion of rotation; while pointing out that in the phage phi29 packaging motor, the genome packaging ATPase of thermophilic viruses (e.g. B204), and FtsK cellular nanobiomotors, the revolution mechanism is applied. Weitao et al. review the structural and functional characteristics of viral and cellular SOS-regulated motor proteins with a focus on the relationship of translocation mechanism to motor function, while Costa et al. discuss the dissolvasome machinery in double Holliday junction dissolution and its molecular mechanism based on the structural interplay between its components. This review illustrates how the molecular mechanisms of Holliday junction related biomotors further differentiates rotation motors (RuvAB, BLM helicases) from revolution motors.

Revolution leads to a thermodynamic edge over rotation, especially in the translocation of lengthy dsDNAs and chromosomes. Due to the length of chromosomes, supercoiling of DNA by biomotors would be a major issue resulting in unnecessary energy consumption. Nature has elegantly evolved a revolution mechanism [4–8] devoid of rotation, torque, and coiling, thus minimizing work terms from molecular friction. The involvement of multiple enzymes in the Holliday junction resolution leads to a complicated motion mechanism. At present, it is controversial whether the Holliday junction dissolvasome motor uses the rotation or revolution mechanism, how many ATP molecules are used, and whether topoisomerases are involved in the DNA translocation process or only at the end of junction resolution [20]. Tackling these questions is challenging due to the many motor components in the dissolvasome.

DNA translocation technology has tremendous potential in a range of biomedical applications, such as the diagnosis and treatment of cancers and viral diseases, as well as high-throughput human genome sequencing. Hopefully, the publication of this issue will be inspiring for the bio- and nanobiotechnology community and will facilitate the application of biomotors.
